# Mechanical and Electrokinetic Effects of Polyamines/Phospholipid Interactions in Model Membranes

**DOI:** 10.1007/s00232-013-9614-z

**Published:** 2013-12-12

**Authors:** Elżbieta Rudolphi-Skórska, Maria Zembala, Maria Filek

**Affiliations:** 1 Institute of Biology, Pedagogical University of Cracow, Podchorążych 2, 30-084 Kraków, Poland; 2 Institute of Plant Physiology, Polish Academy of Sciences, Niezapominajek 21, 30-239 Kraków, Poland

**Keywords:** Phospholipid monolayers, Polyamines, Liposomes’ zeta potential

## Abstract

The mechanical and electrical properties of phospholipids layers influenced by interaction with polyamines were determined by measuring surface pressure and compression modulus of monolayers and zeta potential of liposomes. The saturated derivative of phosphatidic acid (DPPA) formed layers of the organization varying with compression degree. Contact of DPPA layers with polyamines present in the subphase resulted in changing their mechanical properties and the conditions in which the layer reorganization appears. The parameters corresponding to the layer reorganization depended on the size and charge of polyamines’ molecules. The values of: area per DPPA molecule, surface pressure at the point of layer structure reorganization, and surface pressure at the point of collapse characterizing of DPPA layers in the studied systems were determined. It was found that polyamines influenced to a much lesser extent the mechanical properties of monolayers formed from unsaturated derivative of phosphatidic acid slightly increasing its mechanical resistance in the range of higher molecular packing. The results of electrokinetic measurements revealed that surface charge of phosphatidic acid liposomes was effectively neutralized in the presence of polyamines. A similar effect was observed for phosphatidyl glycerol and for negatively charged polystyrene latex particles used as a reference. The influence of polyamines on the mechanical properties of DPPA layers was interpreted assuming a possibility of penetration of the lipid layer by polyamines’ molecules. Comparison of action of putrescine and calcium ions and effects of polyamines on phosphatidyl glycerol provided additional justification for the proposed interpretation of the observed effects.

**Electronic supplementary material:**

The online version of this article (doi:10.1007/s00232-013-9614-z) contains supplementary material, which is available to authorized users.

## Introduction

Phosphatidic acids (PA) are frequently used in physicochemical studies of various phenomena appearing in non-soluble monolayers spread onto aqueous subphases (Lösche and Möhwald 1984; Schalke et al. 2000; Schalke and Lösche 2000). A range of two-dimensional phases can be observed with characteristic phase transitions depending on a detailed lipid structure, subphase composition, and temperature (Demel et al. 1992; Miñones et al. 2002). The layers formed from these molecules are often treated as a model of negatively charged domains in biologic membranes. Like stearic acid for amphiphilics, phosphatidic acids can be regarded as reference substances for comparing surface properties of all class of phospholipids with more complex polar groups. Due to ionic character, interaction of PA with inorganic and organic ions is mainly of electrostatic nature, however, depending on and affecting the layer structure (Lösche and Möhwald 1989; Scott et al. 2003; Vaknin et al. 2003; Faraudo and Travesset 2007a; b).

The role played in living organisms and a broad spectrum of applications stimulate strong interest in determination of the properties and action of the second class of compounds studied in the paper—i.e. polyamines. Polyamines are used in numerous purposes ranging from prevention of plant diseases (Rocha et al. 2005), an increase of plant production (Abd El-Wahed 2006), improvement of stored fruits properties (Liu et al. 2006; Mirdehghan et al. 2007) to various medical applications (Casero and Marton 2007; Agostinelli et al. 2010).

The most important compounds of this group are putrescine (Put), spermidine (Spd), and spermine (Spm). They are present in all compartments of cells, suggesting their involvement in basic cellular processes (Kaur-Sawhney et al. 2003). The concentration of polyamines in plant cells varies in the range 10^−9^–10^−5^ M and far outweighs the amount of hormones (10^−13^–10^−7^ M). Like hormones, polyamines are taking part in the processes of gene expression, membranes stabilization, modulation of enzyme activity, growth, and development of cells (Bachrach 2005; Vladimir et al. 2007). Despite the fact that polyamines synthesized in the cells are so important for various physiological processes, their detailed action is still not fully recovered.

Increased levels of polyamines produced in cells under stress conditions were related to an enhancement of their binding to membranes, preventing cellular damage (Tadolini et al. 1984; Legocka and Kluk 2005). Interaction of polyamines with phospholipids depends on the number of positively charged groups increasing in the sequence: putrescine < spermidine < spermine, and as it was shown by spectroscopic and calorimetric measurements (Bertoluzza et al. 1988), resulting in a modification of the inner structure of the bilayer. Using Langmuir trough technique, it was shown (Gaboriau et al. 2002) that polyamines may penetrate phosphatidic acid layers.

Action of polyamines is regulated by possibility of their transport to and out of the cell. The specific way of polyamine uptaking or excreting by polyamine transport system will be influenced by the changes of cell membrane structure and its properties generated by interaction of membrane lipids with polyamines (Pas) present in and out of cells.

The aim of the paper was to determine quantitatively the changes of mechanical and electrical properties of layers of phosphatidic acids (PA) caused by interaction with polyamines (putrescine, spermidine and spermine) under well controlled conditions. Taking into account the ionic character of the interacting species (PA and Pas), all the measurements were performed for solutions containing constant concentration of inert salt—1 mM KCl (supporting electrolyte) which from one side fixed to some extent the range of electrostatic interactions (ionic strength), on the other created conditions closer to that existing in biologic systems where such ions as K^+^, Na^+^, Ca^2+^ are always present in cytosol and extracellular medium.

Mechanical characteristics of lipid monolayers were investigated using Langmuir trough technique. The effects obtained for PA layers were verified for some other lipids. Electric properties of liposomes (used as a model of bilayer membrane) were expressed by zeta potentials values which were compared with data obtained for negatively charged polystyrene latex particles (taken as a reference system of well defined negatively charged particles).

Action of polyamines was compared to the effects of calcium ions. This comparison allows better understanding of the role of these agents in biologic systems, in particular, their importance for the stabilization of cell membranes. Calcium ions are considered as factors that balance electrostatics of bio-membranes. Putrescine, present in cells at relatively high concentrations, may enhance the action of calcium ions under conditions where calcium ions are involved in other life processes, or when under stress conditions there is their deficiency. Stabilizing effect of polyamines involving the modification of the electrical state of membranes by favoring the maintenance of homeostasis can protect bio-membranes against damage by reactive oxygen species that appear under peroxidative conditions. Therefore, determination of the physicochemical parameters of systems mimicking bio-membranes may help understanding of the defense mechanisms that nature has provided the living organisms.

## Materials and Methods

### Materials

Synthetic lipids: PA—1,2-dipalmitoyl-sn-glycero-3-phosphate 16:0 (DPPA) and 1,2-dioleoyl-*sn*-glycero-3-phosphate 18:1 (DOPA). 1,2-Dipalmitoyl-*sn*-glycero-3-phosphocholine 16:0 (DPPC) and 1,2-dipalmitoyl-*sn*-glycero-3-phospho-(1′-*rac*-glycerol) 16:0 (DPPG) were obtained from Sigma-Aldrich. 1,2-Dioleoyl-*sn*-glycero-3-phospho-(1`-*rac*-glycerol) 18:1 (DOPG) was from Avanti Polar Lipids (USA/Canada).

 Polystyrene latex suspensions were synthesized by emulsion polymerization using potassium persulfate as an initiator according to the method described (Goodwin et al. 1974). Reaction product was carefully purified by steam distillation and ultrafiltration.

Polyamines: putrescine (Put), spermidine (Spd), and spermine (Spm) were purchased from Sigma-Aldrich.

KCl and HCl used in experiments were of chemical purity.

Solvents (chloroform, methanol) of chemical purity were from POCh (Poland).

Freshly purified water was produced by HLP 5 apparatus Hydrolab (Poland) by ion-exchange and reverse osmosis.

1 mM KCl was used as a supporting electrolyte in all experiments.

As Pas strongly increased pH, Pas solutions of concentrations higher than 10^−4^ M were neutralized by HCl addition to keep pH around 7.

### Methods

#### Surface Pressure Isotherms

Surface pressure isotherms were obtained using Langmuir trough (Minitrough, KSV, Finland) of total surface area 243 cm^2^ with Pt-Wilhelmy plate used for surface tension detection. Lipids were spread onto subphase and surface pressure isotherms were taken after 15 min waiting period at constant rate of compression equal to 5 mm/min at temp. 25^o^ C. The directly measured isotherms presented as a dependence of surface pressure *π* versus area per lipid molecule *A*_m_ were differentiated for obtaining static compression modulus

$$C_{\text{s}}^{ - 1} = - \frac{d\pi }{{d{ \ln }A_{\text{m}} }}$$

#### Electrokinetic Measurements

Liposomes from studied lipids were produced according to the standard procedure. Thin layer of lipid was formed by evaporating (under argon) of the lipid solution (in chloroform or chloroform/methanol mixture) wetting the walls of the round-bottomed glass cell. Evaporation procedure was continued until constant weight of lipid sample was achieved. Next, a defined amount of 1 mM KCl solution of temperature above lipid phase transition was added to the cell with a lipid film and the whole mixture was subjected to ultrasonification and vortexing. Such prepared liposomes have mean sizes (as ascertained by dynamic light scattering (DLS) method) in the range between 200 and 600 nm. Suspensions of freshly prepared liposomes were used in zeta potential determination. Liposomes’ electrophoretic mobility was determined by dynamic light scattering technique using Malvern Zetasizer ZS apparatus. The values of mobility were converted to zeta potentials using Smoluchowski’ equation.

The effect of Pas presence was determined by mixing the liposomes’ suspensions with appropriate amount of Pas solutions (neutralized with HCl when necessary) in 1 mM KCl.

The effect of polyamines on zeta potential was determined also for monodisperse polystyrene latex particles (800 nm in diameter) negatively charged due to the presence of strong sulfate groups chemically incorporated in the polystyrene polymer chain. This system can be treated as model for estimation of a negative charge compensation by Pas not complicated by a possibility of counter-ion penetration into a particle.

## Results

### Surface Pressure Isotherms and Compression Modulus

**Fig. 1:**
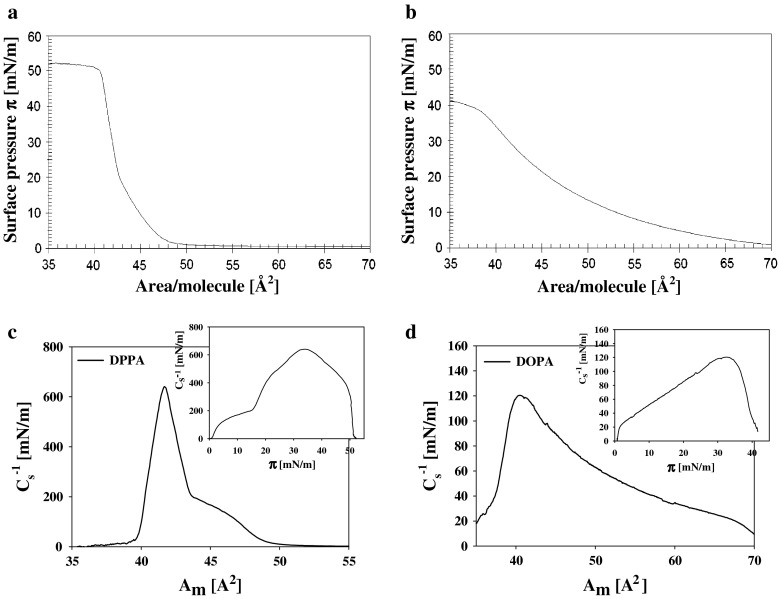
Surface pressure isotherms (*π* vs. *A*_m_) of DPPA (16:0) (**a**) and DOPA (18:1) (**b**) layers on a supporting electrolyte (1 mM KCl). Static compression modulus $C_{\text{s}}^{ - 1}$ vs. area per molecule *A*_m_ for the same systems: DPPA (16:0) (**c**) and DOPA (18:1) (**d**). (Insets present the dependencies of $C_{\text{s}}^{ - 1}$ on surface pressure ***π***)

Surface pressure isotherm of DPPA on supporting electrolyte has a characteristic shape with a break point corresponding to strictly defined packing of the molecules at which the rate of pressure increase (with the degree of compression) is changing. The breaking point of isotherm is identified with the two-dimensional phase transition within the lipid layer related to a modification of layer organization. The isotherm of phosphatidic acid derivative containing unsaturated fatty acid residues—DOPA (18:1) do not exhibit this characteristic behavior (Fig. 1a, b).

The specific features of surface pressure isotherms are more clearly visible on the values of compression modulus presented as a function of either area per lipid molecule $C_{\text{s}}^{ - 1} = f\left( {A_{\text{m}} } \right)$ (Fig. 1c, d) or layer surface pressure $C_{\text{s}}^{ - 1} = f\left( \pi \right)$ (insets to Fig. 1c, d). $C_{\text{s}}^{ - 1} = f\left( {A_{\text{m}} } \right)$ dependence for DOPA layers show that, generally small values of $C_{\text{s}}^{ - 1}$ increase with density of molecules (with a decrease of area per molecule) till the point where the whole layer collapse due to further reduction of the total surface area restricted by approaching barriers. The maximal value of $C_{\text{s}}^{ - 1}$ for DOPA was only 120 mN/m. For DPPA this quantity reaches values above 600 mN/m.

**Fig. 2:**
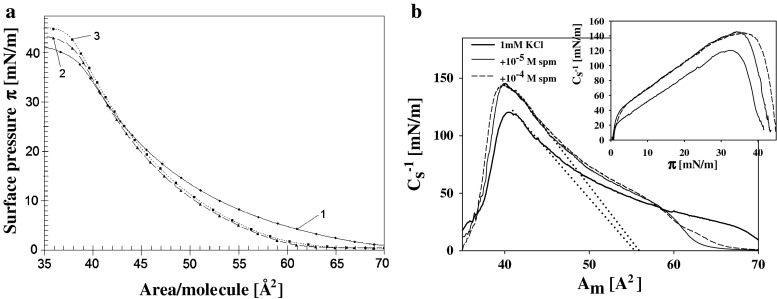
**a** Surface pressure isotherms of DOPA (18:1) layers on: 1 mM KCl (*solid line*) (*1*), 1 × 10^−5^ M spermine in 1 mM KCl (*long dash line*) (*2*) and on 1 × 10^−4^ M spermine in 1 mM KCl (*short dash line*) (*3*). **b** Static compression modulus $C_{\text{s}}^{ - 1}$ vs. area per molecule *A*_m_ for the same systems. (Inset presents the dependence of $C_{\text{s}}^{ - 1}$ on surface pressure ***π***)

The presence/absence of unsaturated fatty acid residues in the lipid molecules influences also the effect of polyamines onto mechanical properties of their monolayers. Addition of spermine (of highest positive charge) to a subphase modifies mechanical properties of DOPA layer in the lower pressure range (between 0 and 20 mN/m) (Fig. 2a). Static compression modulus reveals also differences (less clearly displayed on surface pressure isotherms) appearing in the range of higher pressures (lower values of area per molecule (Fig. 2b)). (The effects of less active spermidine and putrescine on DOPA layers were smaller).

**Fig. 3:**
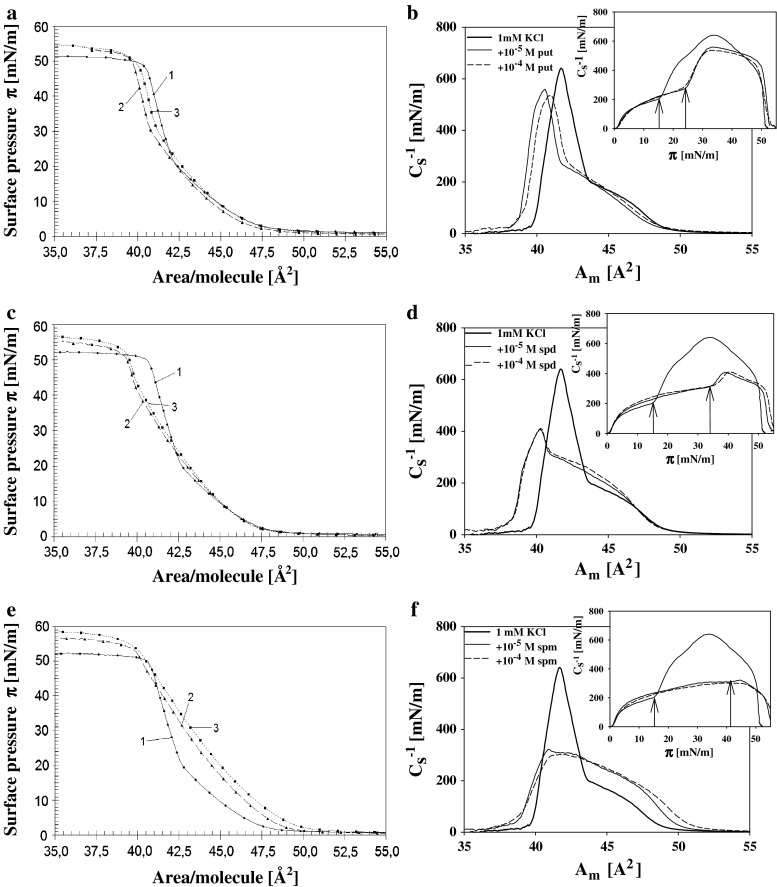
**a**, **c**, **e** Isotherms of surface pressure *π* vs. area per DPPA molecule of layers on 1 mM KCl (*solid lines*) (*1*), on electrolyte containing 1 × 10^−5^ M: putrescine **a**, spermidine **b** and spermine **c** (*long dash lines*) (*2*) and on subphases containing 1 × 10^−4^ M of the same Pas—*short dash lines* (*3*). **b**, **d**, **f** Static compression modulus $C_{\text{s}}^{ - 1}$ vs. area per molecule ***A***_**m**_ for the same systems. (Insets present the dependencies of $C_{\text{s}}^{ - 1}$ on surface pressure ***π***)

Mechanical properties of DPPA layers were more significantly changed by the presence of polyamines in a subphase (Fig. 3). The effects caused by studied Pas were practically the same for polyamines additions at concentrations equal to 10^−5^ and 10^−4 ^M. In the presence of polyamines the point of phase transition, which for the supporting electrolyte was observed at *π* = 15 mN/m, was shifted to higher pressures which exact values depended on the number of cationic groups in Pas. Putrescine, containing two amino groups, affected the properties of DPPA monolayers to the slightest degree, displacing the break point of the isotherm by about 10 mN/m, while spermine, with four cationic groups moved this point up by above 25 mN/m. (These differences were more pronouncedly visible on graphs representing the dependence of compression modulus $C_{\text{s}}^{ - 1}$ on *π* (insets in Fig. 3b, d, f).) It is also interesting to compare the dependencies of compression modulus on *A*_m_—area per DPPA molecule for studied systems. (Fig. 3b, d, f). One can see that maximal value of $C_{\text{s}}^{ - 1}$ reached at higher packing density of the DPPA layer on pure supporting electrolyte was only a little higher in comparison to the values obtained in presence of putrescine but this difference increases meaningfully for spermidine and spermine.

The presence of Pas in a subphase also affects the collapse pressure of PA layers which value increases when comparing to the value characteristic for layers on pure 1 mM KCl. (Collapse pressure was determined as the intersection of the lines extrapolating surface pressure isotherm in the range of dense molecular packing and the pressure of the layer after collapse.) In the case of DPPA layer formed on the 1 mM KCl collapse occurs at the pressure of 50 mN/m while on the subphases containing putrescine, spermidine and spermine at *π* equal to 51, 52 and 54 mN/m, respectively. Corresponding values for DOPA were: 38 (supporting electrolyte), 39, 42, and 42 mN/m for putrescine, spermidine, and spermine, respectively.

In preliminary measurements of the surface activity of the polyamines’ solutions (10^−5^ and 10^−4^ M) used as subphases for lipid layers it was found that no decrease of the surface tension values was observed.

The influence of barrier speed onto surface pressure isotherms was also checked. Isotherms taken at compression rates equal to 2.5, 5, and 10 mm/min were the same within experimental error (see Fig. S1 in Supplementary material). This and the negligible surface pressure hysteresis indicate that systems were close to equilibrium under studied conditions.

**Fig. 4:**
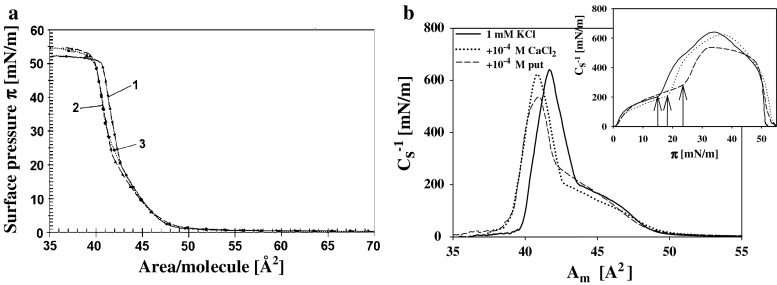
**a** Isotherms of surface pressure *π* vs. area per DPPA molecule for layers deposited on 1 mM KCl (*solid line*) (*1*); on supporting electrolyte containing 1 × 10^−4^ M CaCl_2_ (*dash line*) (*2*) and on subphase containing 1 × 10^−4^ M of putrescine (*short dash lines*) (*3*). **b** Static compression modulus $C_{\text{s}}^{ - 1}$ vs. area per molecule *A*_m_ for the same systems. (Inset presents the dependencies of $C_{\text{s}}^{ - 1}$ on surface pressure ***π***)

To answer the question whether the size and nature of counterions affects the properties of DPPA layers, the surface pressure isotherms were detected for DPPA spread on subphase containing calcium ions of the same charge as putrescine. It was found that 10^−4 ^M calcium ions produces qualitatively similar but little smaller effects: the isotherm break points at the surface pressure isotherms were shifted to higher values by 3 and 9 mN/m and the maximal values of compression modulus were 620 and 537 mN/m for calcium and putrescine, respectively (compare Fig. 4a, b).

In Table 1 the main parameters of surface pressure isotherms influenced by Pas were collected.

**Table 1:** Main parameters of surface pressure isotherms for phosphatidic acids (PA) monolayers on subphases without and with additions of Pas

System	Area/PA molecule *A*_m__ at dense packing_ (Å^2^)	Surface pressure *π*_break_ (mN/m)	Collapse surface pressure *π*_collapse_ (mN/m)
DOPA on 1 mM KCl			38
DOPA + 1 × 10^−5^ putrescine			38
DOPA + 1 × 10^−4^ putrescine			40
DOPA + 1 × 10^−5^ spermidine			41
DOPA + 1 × 10^−4^ spermidine			43
DOPA + 1 × 10^−5^ spermine			41
DOPA + 1 × 10^−4^ spermine			43
DPPA on 1 mM KCl	43.6	15	50
DPPA + 1 × 10^−5^ putrescine	41.6	24	50
DPPA + 1 × 10^−4^ putrescine	42	23	51
DPPA + 1 × 10^−5^ spermidine	41	34	50
DPPA + 1 × 10^−4^ spermidine	41	34.5	52
DPPA + 1 × 10^−5^ spermine	~41	~43	54
DPPA + 1 × 10^−4^ spermine	~41	~43	55
DPPA + 1 × 10^−4^ CaCl_2_	42.7	18	55

**Fig. 5:**
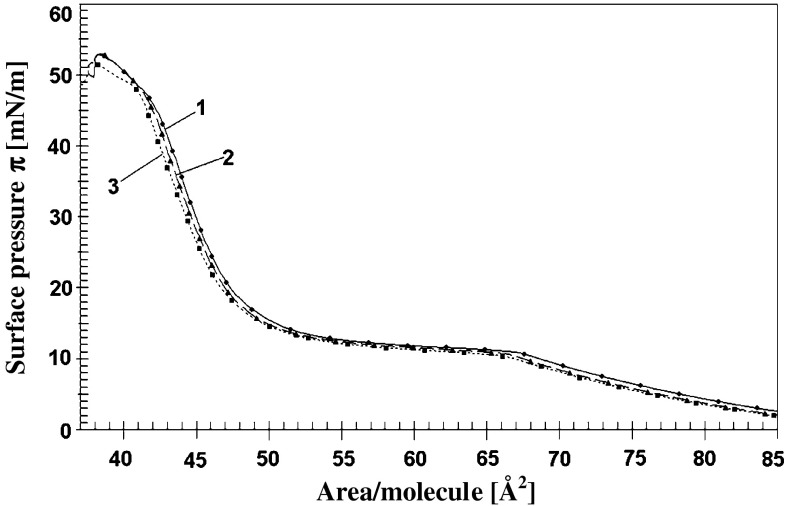
Isotherms of surface pressure *π* vs. area per DPPC molecule *A*_m_ for a layer on 1 mM KCl (*solid lines*) (*1*), on electrolyte containing 1 × 10^−5^ M spermine (*long dash lines*) (*2*) and on subphase containing 1 × 10^−4^ M of the same Pas (*short dash lines*) (*3*)

To determine the reaction of other lipids to the presence of polyamines some measurements were performed with phosphatidyl-choline and phosphatidyl-glycerol. Surface pressure isotherms (Fig. 5) of DPPC layers onto pure supporting electrolyte and in the presence of highly charged spermine cations did not differ from each other.

**Fig. 6:**
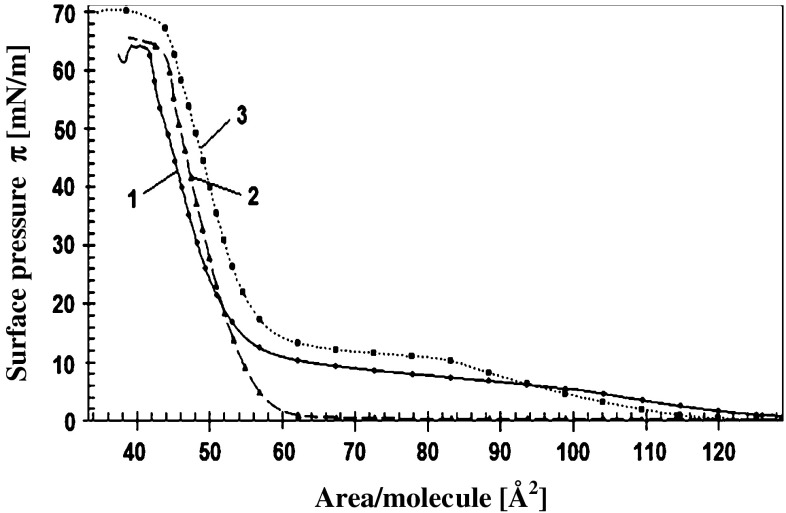
Isotherms of surface pressure vs. area per DPPG molecule *A*_m_ for layers deposited on 1 mM KCl (*solid line*) (*1*); on supporting electrolyte containing 1 × 10^−4^ M CaCl_2_ (*dash line*) (*2*) and on subphase containing 1 × 10^−4^ M of putrescine (*short dash lines*) (*3*)

Comparison of properties of DPPG layers spread onto supporting electrolyte and onto electrolyte containing 10^−4 ^M CaCl_2_ or 10^−4^ M putrescine shows that calcium counterions modify DPPG surface pressure isotherms in the range of low molecular packing causing the disappearance of LE–LC transition (present on pure electrolyte) and small increase of isotherm slope in the range of condensed state. The presence of putrescine at the same concentration leads to an increase of the values of surface pressure corresponding to the phase transition and to the point of layer collapse (Fig. 6).

### Electrokinetic Measurements

**Fig. 7:**
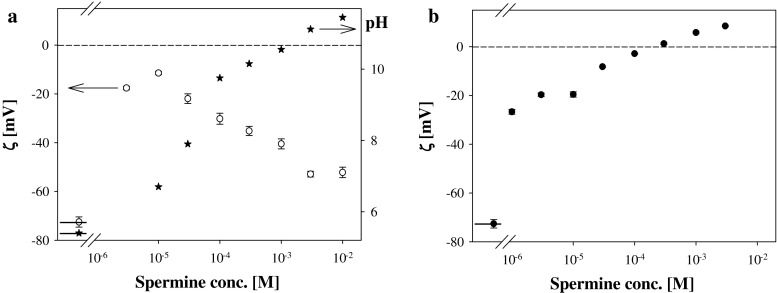
The dependence of zeta potential of latex particles on spermine concentration. **a** Spermine solutions of natural pH, which values are represented by stars. **b** Spermine solutions neutralized by an addition of HCl to reach pH close to 7. Short sections on the left side of graphs represent the zeta potential values measured in pure supporting electrolyte (1 mM KCl)

Zeta potential values of latex particles in solutions containing spermine of various concentrations are presented at Fig. 7. Part a presents the dependence of zeta potential on spermine concentration when solutions were not neutralized by HCl addition. pH values of non-neutralized Spm solutions increased dramatically with spermine concentration reaching the value of about 11 for 10^−2^ M Spm. Such pH increase resulted in non-monotonical change of latex zeta potential which value, after initial increase, started to decrease (for Spm concentrations higher than 3 × 10^−5^ M) being negative in all studied spermine concentrations. (Similar pH effect on zeta potential values was observed for liposomes made from studied lipids). Part b presents results of latex zeta potentials as a function of spermine concentration when solutions were neutralized by addition of an appropriate amount of HCl. Under such conditions the values of zeta potential increased monotonically with Spm concentration and sign reversal was observed for Spm content higher than 10^−4^ M. To avoid simultaneous changes of Pas concentrations and pH all further presented results were obtained for neutralized Pas solutions.

**Fig. 8:**
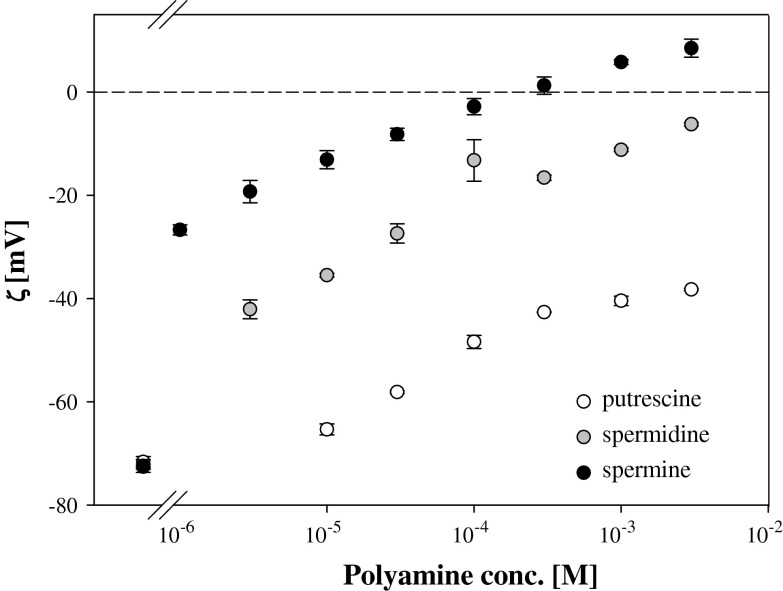
The dependence of zeta potential of latex particles on Pas concentration for solutions neutralized by HCl addition: putrescine (*open circle*), spermidine (*grey circle*), spermine (*black circle*). *Short section* on the left side represents the latex zeta potential in 1 mM KCl

According to expectations the efficiency in surface charge compensation increased with number of amino groups in Pas molecules being the highest for spermine and lowest for putrescine (Fig. 8). The results obtained for these three compounds show that in case of spermine, zeta potential of latex particles reversed its sign whereas neither spermidine nor putrescine, despite increasing zeta potential values, did not cause the charge reversal in studied concentration range under studied conditions.

**Fig. 9:**
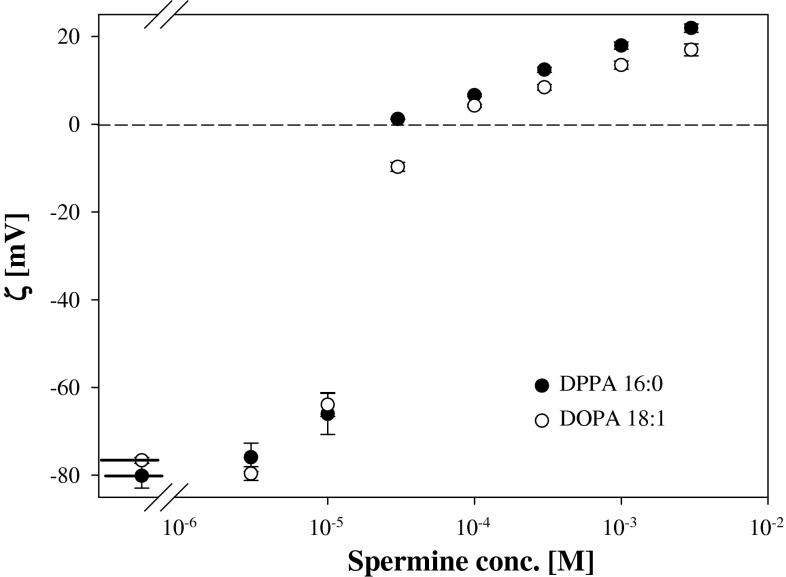
The dependence of zeta potential of DPPA (16:0) (*black circle*) and DOPA (18:1) (open circle) on spermine concentration. *Short sections* on the left side represents the zeta potential values of liposomes in 1 mM KCl

It was found that electrostatic effects found for lipids of identical polar groups but having saturated and unsaturated fatty acid residues were very similar. The dependence of zeta potentials of liposomes prepared from DPPA (16:0) and DOPA (18:1) on spermine concentration demonstrates this observation (Fig. 9).

According to expectations and similar to the effects detected for latex particles, the ability for compensation of the negative charge of phosphatidic ions increases with number of amino groups in Pas molecules in series: spermine > spermidine > putrescine (Fig. 8). In the studied Pas concentration range (3 × 10^−6^ to 3 × 10^−3^ M) spermine cations were able to overcharge phosphatidic layer. In case of spermidine, liposomes’ surface charge was hardly compensated at highest studied concentration (3 × 10^−3^ M).

**Fig. 10:**
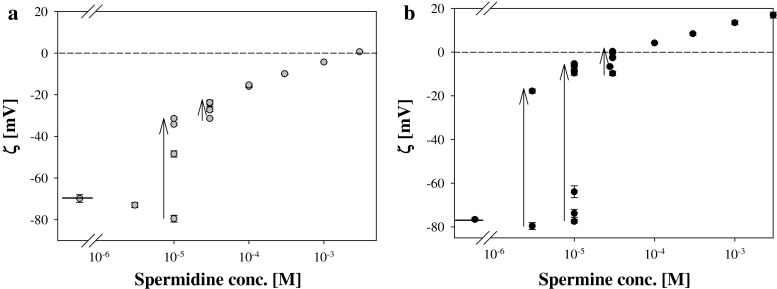
The dependence of zeta potential of DOPA (18:1) liposomes on concentrations of spermidine (**a**) and spermine (**b**). *Arrows* represent the direction of the change of zeta potential values when liposomes’ suspensions were diluted by Pas solution of the same concentration. *Short sections* on the left graph sides represent the zeta potential values of liposomes in 1 mM KCl

It is worth to notice that in the region of low spermine and spermidine concentrations (below 10^−4 ^M) the results of liposomes zeta potentials depended on liposome particle concentration in suspension. The lower content of liposomes’ particles the higher value of zeta potential was detected. This effect was presented in Fig. 10, where arrows show the change in zeta potential values when concentration of liposome’ particles was decreased (by 2, 4, 8…times dilution by studied solution along the direction indicated by an arrow). Such an effect was not observed for polystyrene latex suspensions despite that spermidine and spermine produced comparable changes of the particles’ zeta potentials within studied Pas concentration range.

**Fig. 11:**
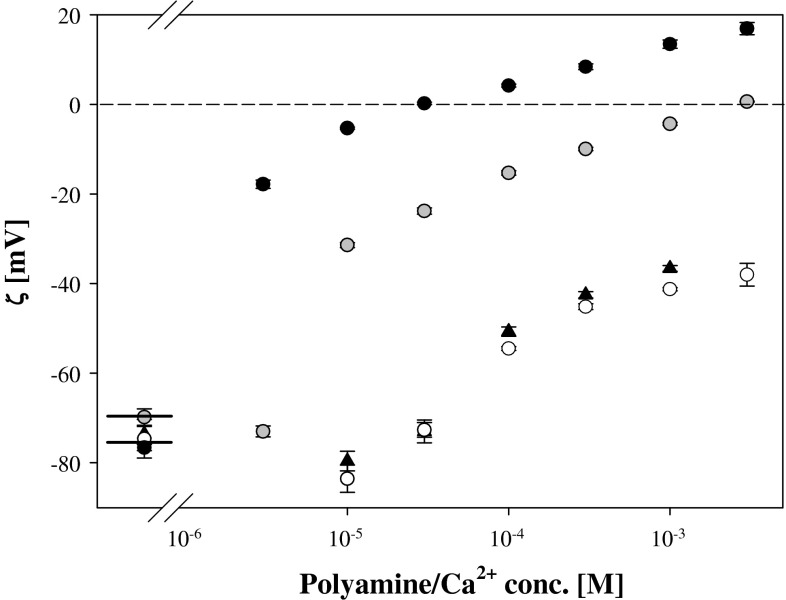
The dependence of zeta potential of DOPA (18:1) liposomes on concentration of: spermine (*black circle*), spermidine (*grey circle*), putrescine (*open circle*), and CaCl_2_ (*black triangle*). *Short sections* on the left side of graph represent the liposomes zeta potential values in 1 mM KCl

DOPA liposomes exhibited practically identical values of zeta potentials in solutions containing the same concentrations of putrescine or calcium cations (Fig. 11).

**Fig. 12:**
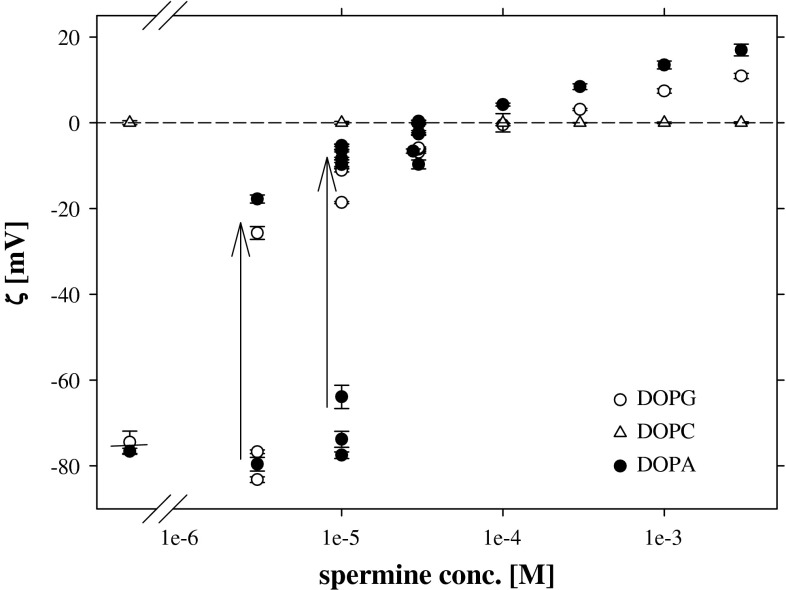
The dependence of zeta potential of DOPA (18:1) (*black circle*), DOPG (*open circle*), and DOPC (*open triangle*) liposomes on spermine concentration. *Arrows* represent the direction of the change of zeta potential values when liposomes’ suspensions were diluted by spermine solution of the same concentration. *Short sections* on the left graph sides represent the zeta potential values of liposomes in 1 mM KCl

Negative surface charge of liposomes formed from DOPG was neutralized by spermine in a very similar way as it was found for negatively charged polystyrene latex particles and PA liposomes (including the effect of liposome particle concentration). For neutral PC liposomes the presence of the most active spermine did not induce any changes of zeta potential (Fig. 12).

## Discussion

As can be seen in Fig. 3 the mechanical properties of DPPA layers are strongly modified in Pas presence. Very high values of $C_{\text{s}}^{ - 1}$ for DPPA layer in its condense state on pure supporting electrolyte are strongly diminished when the layers were formed onto subphases containing relatively low Pas concentrations (10^−5^, 10^−4^ M). The reduction degree increases in order: putrescine < spermidine < spermine. The values of compression modulus characterizing the mechanical resistance against compression are related to packing density and molecular interactions within the layer. Thus, high values of this parameter are characteristic for densely packed films in which strong interactions are able to counteract their compression and much smaller for less ordered two-dimensional gaseous and liquid-like layers. Dramatic change of mechanical properties of DPPA layers caused by organic counterions can result from modification of composition and thereby interactions within a layer. It is worth to notice that spermine at concentration 10^−5^ M and spermidine of 10^−4 ^M—conditions under which the zeta potentials of PA liposomes are practically identical – induce different changes in the mechanical properties of DPPA layers. This observation gives support for linking the observed effects with the Pas penetration. (Possible time dependence of incorporation of Pas molecule into lipid layer was checked in separate experiments where DPPA layer compressed to the surface pressure equal to 45 mN/m was left for 5 or 30 min in contact with subphase containing 10^−5^ M spermine and next the whole cycle expansion/compression was repeated. No noticeable differences in surface pressure isotherms were found for these two contact times—see Fig. S2 in Supplementary material).

Comparison of the action of putrescine and calcium ions shows that although the electrostatic effects caused by these factors are virtually identical (Fig. 11), both affected the mechanical properties of the DPPA layer somewhat differently, with Ca^2+^ cations producing smaller changes. (Fig. 4a, b and Table 1). This result confirms conclusions made on the basis of molecular dynamic simulations (Casares et al. 2008) that the effect of inorganic cations on the properties of the DMPA monolayer in its solid phase can be neglected. However, in other studies lipid layer reorganization and inorganic cation insertion into lipid layer was considered (Lösche and Möhwald 1989; Kaznessis et al. 2002). For the system: PA/Ba^2+^ an excess adsorption of ions was assigned to their interaction with oxygen atoms in glycerol backbone of PA molecule (Vaknin et al. 2003; Faraudo and Travesset 2007b). Strong experimental support for this idea comes from noticing that overcharging by divalent counterions is not observed for layers of simple fatty acid. In Martín-Molina et al. (2010) experimental and simulation evidence for strong interconnection of charge reversal and ions’ capability for soft layer penetration was presented.

The mechanism of interaction of counter-ions with charged lipid layers is related to the problem of charge distribution within the lipid polar group region, the structure of lipid mono/bi-layer and possible interference coming from interfacial and hydration water. These problems were considered in many papers where molecular dynamic simulations were used for modeling the system: charged lipid layers/counterions (Faraudo and Travesset 2007b; Casares et al. 2008; Kaznessis et al. 2002; Martin-Molina et al. 2012; Faraudo 2011). The results of calculations obtained for various lipids and various inorganic counterions give some ideas about effects expected for macroscopic mono- and bi-layers. However, simulations are carried out for idealized homogeneous systems of limited size whereas BAM pictures (Miñones et al. 2002; Berdycheva et al. 2003; Miñones et al. 2003; Grigoriev et al. 2003) of macro-size lipid layers reveal their significant heterogeneity. Thus one cannot directly transfer theoretical predictions onto experimentally measured quantities, especially that related to mechanical properties of macroscopic layers.

Comparison of the results obtained for the systems: DPPA + Put, DPPA + Ca (Fig. 4), and DPPG + Put, DPPG + Ca (Fig. 6) clearly shows significant role played by lipid layer structure and accessibility of negative charge located within lipid polar group. Already properties of layers of DPPG and DPPA on pure electrolyte are different with characteristic inflection on the DPPG surface pressure isotherm at low pressure values (absent in case of DPPA) and much higher maximal values of $C_{\text{s}}^{ - 1}$ (at high molecular packing) for DPPA than for DPPG. It is interesting to notice that Ca and Put counterions produce very similar changes of zeta potentials of PA and PG liposomes (Figs. 11, 12). Otherwise, significant differences in the effects of these ions on the mechanical properties of the DPPA and DPPG layers (as can be seen on the surface pressure isotherms Figs. 4, 6) were found. In presence of calcium cations the characteristic inflection on the DPPG surface pressure isotherm disappears and some increase of compression modulus of the layer at higher molecular packing can be noticed. This last observation agrees with the data of Stevanato et al. (1997) who observed an increase in rigidity of DMPG bilayer in contact with Ca^2+^.

Putrescine practically did not influence the DPPG layer properties at condense state ($C_{\text{s}}^{ - 1}$ values) but caused slight increase of values of surface pressure corresponding the LE–LC coexistence and collapse pressure indicating layer stability.

The system: DPPG/Pas was studied by Berdycheva et al. (2003) who found that polyamines cause meaningful increase of mean area per DPPG molecule. The Brewster angle microscopic (BAM) pictures presented in the paper demonstrate large heterogeneity of the layer thus the observed changes in *A*_m_ should not be identified with molecular area of lipid molecule in a homogeneous monolayer. The results of infrared reflection absorption spectroscopy (IRRAS) studies allowed authors to conclude that Pas (putrescine and spermine) stay adsorbed on the monolayer without insertion.

Full access of phosphate group to all components of polar subphase in case of PA layers makes interaction with counterions free from steric constraints created by an additional group bound to the phosphate charge center as it is in the case of PG. The results of surface pressure (Garidel and Blume 2005) and of infrared spectroscopy (Garidel et al. 2000) demonstrated a key role played by polar group conformation in binding of counterions to PG (what explains also the differences of effects caused by counter ions of various sizes).

The geometric matching of layer components and interacting species seems to be extremely important for the changes of layer structure and thereby its mechanical properties. The layers of unsaturated lipid—DOPA are less organized due to steric obstacle created by the presence of double bound in fatty acid residues. The less packed structure of unsaturated lipid layers influences interactions within a layer and with substances present in a subphase and, consequently, the mechanical properties of the layer (Ichimori et al. 1999; Murzyn et al. 2001; Hąc-Wydro et al. 2009). In presence of Pas, mechanical properties of DOPA layers are only slightly modified despite strong electrostatic interaction. The action of most active spermine causes a decrease in $C_{\text{s}}^{ - 1}$ in the range of low layer density due to a decrease of electrostatic repulsion between DOPA anions (Fig. 2). In the range of higher density of DOPA molecules mechanical resistance of the layer in the presence of spermine slightly increases. The assumption of spermine penetration into DOPA layer can qualitatively explain an increased value of compression modulus. As there is no significant change in the area per DOPA molecule in a layer of this lipid onto supporting electrolyte and in the presence of spermine thus one can conclude that observed increase in resistance to compression can be attributed to changes in the interactions within the mixed layer.

Observed growth of the values of the collapse pressure of PA layers in presence of longer Pas in the subphase (Table 1) points to stabilizing effect of these substances.

Analysis of the results has shown that electrostatic effects induced by the interaction between polyamines and phosphatidic ions are far less specific as compared to changes in the mechanical properties of the PA monolayers where molecular organization and van der Waals interactions contribute to the final effect. Zeta potential values were not sensitive to the presence/absence of double bonds in fatty acid residues in PA molecules giving practically identical dependencies for DPPA and DOPA liposomes on Pas concentrations.

Non-monotonic dependence of zeta potential values of latex particles and liposomes in not neutralized solutions on Pas concentration (Fig. 7a) indicates the strong influence of pH factor on the properties of studied systems. On the one hand, a strong increase in pH value (with Pas content) leads to a decrease of Pas ionization degree, on the other to an increase of PA ionization and probable adsorption of OH^−^ ions. Dramatic effect of pH points to the role played in living organisms the genetically determined cell homeostasis that provides the correct course of all life processes (Kay et al. 1986).

Under conditions of maintained constant solution acidity electrostatic interaction of Pas cations with phosphatidic ions results in negative charge neutralization. The charge neutralization seems to be typical for phospholipids with negatively charged polar groups as indicated by almost the same dependencies of zeta potentials of PA and PG liposomes on spermine concentration.

The only particular electrostatic effect noticed for liposomes formed from both: DOPG and DOPA is a strong dependence of zeta potential values on the content of liposome particles in suspension, perceptible in the range of low (<10^−4^ M) concentrations of spermidine and spermine (Figs. 10, 12). No such effect was found for latex particles. This means that it should be linked to the structure of the liposome particle (different from that of latex). Liposome particle’ design may allow embedding Pas molecules deeper into lipid bilayer. Such assumption can explain the depletion of Pas molecules observed at higher liposome number concentration. Only at low liposome particle concentration and at higher polyamines contents the stock of Pas molecules in solution is sufficient for both penetration and adsorption. Unfortunately, dynamic light scattering technique used for electrophoretic mobility and liposome size determination does not allow to estimate an effective area of particles which value is necessary for comparison of the number of Pas molecules in solution in relation to the number of active adsorption centers at liposome surface. However, despite the uncertainty related to this value it is worth to notice, that at least spermine, of largest Pas molecule, compensate PA liposomes charge slightly more effectively than that of latex particles (both objects exhibited very similar zeta potential values in pure supporting electrolyte solution).

Neither electrical state nor mechanical properties of layers formed from neutral lipids like PC react to the presence of polyamine cations. Thus electrostatic interaction seems to be prerequisite initial condition for consecutive layer reorganization. This observation agrees with the results of Bertoluzza et al. (1988) obtained from Raman spectroscopy. Momo et al. (1995) reported some changes in structure of phosphatidylcholine liposomes after contact with spermine, however they used choline of natural origin (egg-yolk) and rather concentrated (0.1 M) Hepes buffer thus these conditions cannot be compared with ours. Results presented in the later work of these authors (Momo et al. 2000) obtained for liposomes formed from DMPC showed that polyamines practically did not associate to DMPC whereas they effectively affect the structure of negatively charged DMPG bilayer changing its transition temperature.

Presented results may be helpful for understanding the bio-processes taking place in cells, which functions are influenced by polyamines present on both sides of cell membranes.

## Conclusions

The surface pressure isotherms and zeta potential measurements for PA showed that electrostatic interactions with Pas of increasing size and charge leads to significant changes of mechanical properties of PA layers and effective charge compensation of liposomes. When comparing the effect of Pas present in the subphase at low concentrations on properties of DPPA and DOPA it was found that despite similar compensation of charge compressibility of densely packed DPPA layers (expressed by static compression modulus) was significantly increased (rigidity decreased) whereas opposite effects were observed for layers of DOPA (containing unsaturated fatty acid residues). These observations can be explained by assuming lipids’ layers penetration by Pas molecules which in case of saturated derivative leads to disorganization of highly ordered layer structure, whereas for unsaturated lipid to an increase of layer stiffness.

The influence of equally charged putrescine and calcium ions supports postulated explanation with Ca^2+^ modifying DPPA layer structure to smaller extent than Put.

Properties of DPPA and DPPG layers being different already on pure supporting electrolyte were otherwise modified by Pas and Ca presence. Glycerol group in DPPG molecule may create a steric obstacle for penetration of larger ions and consequently the differences in effects caused by Ca and putrescine onto DPPG layer are much larger than for DPPA.

The increase in collapse pressure indicates a stabilizing effect of polyamines on the layers of examined negatively charged phospholipids (DPPA, DOPA, and DPPG).

Neither mechanical nor electric properties of neutral phosphatidylcholine layers were changed in presence of studied cations proving that electrostatic interaction seems to be prerequisite initial condition for consecutive layer reorganization.

## Electronic supplementary material

Below is the link to the electronic supplementary material.

Supplementary material 1 (DOCX 220 kb)
