# Supplementary material for: Mechanical and Electrokinetic Effects of Polyamines/Phospholipid Interactions in Model Membranes
Source: J Membr Biol. 2013 Dec 12;247(1):81–92. doi: 10.1007/s00232-013-9614-z (PMC3889835; doi:10.1007/s00232-013-9614-z)
Supplement: Supplementary file 1 — Supplementary material 1 (DOCX 220 kb) [file 232_2013_9614_MOESM1_ESM.docx]

Supplementary material to the Manuscript: “**Mechanical and electrokinetic effects of polyamines/phospholipid interactions in model membranes.”**

by Elżbieta Rudolphi-Skórska, Maria Zembala, Maria Filek

Fig. S1. Surface pressure isotherms (***π*** vs. ***A_m_)*** of DPPA (16:0) spread on pure supporting electrolyte (1 mM KCl) - 1 (solid line); on subphase containing 10^-5^ M spermine - 2, 3, 4 taken at barrier speeds equal to: 2.5 mm/min (dotted line); 5 mm/min (short dash line) and 10 mm/min (long dash line).

Fig. S2. Surface pressure isotherms (***π*** vs. ***A_m_)*** of DPPA (16:0) spread on subphase containing 10^-5^ M spermine taken at barrier speed equal to 5 mm/min. Layers were first compressed to surface pressure equal to 45 mN/min and then reversed after waiting time equal to: 0 - (dotted line); 5 min (short dash line) and 30 min (long dash line).
